# Application of biomarkers in the diagnosis of kidney disease

**DOI:** 10.3389/fmed.2025.1560222

**Published:** 2025-04-30

**Authors:** Zuohua Lu, Weifeng Ni, Yuding Wu, Bin Zhai, Qiuyun Zhao, Tian Zheng, Qianqian Liu, Dapeng Ding

**Affiliations:** ^1^Department of Clinical Laboratory, Gongli Hospital of Shanghai Pudong New Area, Shanghai, China; ^2^Department of Endocrinology, Rheumatology and Immunology, The Second Affiliated Hospital of Shantou University Medical College, Shantou, China; ^3^Goldsite Diagnostics Inc., Shenzhen, China; ^4^Department of Clinical Laboratory, Baotou Central Hospital, Baotou, China; ^5^Department of Clinical Laboratory, Guilin Hospital of Integrated Traditional Chinese and Western Medicine, Guilin, China; ^6^Department of Clinical Laboratory, Jinling Hospital, Affiliated Hospital of Medical School, Nanjing University, Nanjing, China; ^7^Department of Clinical Laboratory, First Affiliated Hospital of Dalian Medical University, Dalian, China

**Keywords:** biomarker, kidney disease, chronic kidney disease, CKD, acute kidney disease, AKD, kidney injury, biomarker combination

## Abstract

Worldwide, kidney disease has grown to be an important global public health agenda that reduces longevity. Medical institutions around the globe should enhance screening efforts for kidney disease, to facilitate early kidney disease detection, diagnosis, and intervention. Common screening methods for nephropathy encompass renal tissue biopsy, urine dry chemistry tests, urine formed element analysis, and urine-specific protein assays, among others. These methodologies evaluate renal health by scrutinizing a spectrum of biomarkers. Precise classification and quantitative analysis of these biomarkers can assist in determining the site and extent of kidney injury, as well as in assessing treatment efficacy and prognosis. In this paper, we reviewed the methods and biomarkers for kidney disease and also the integration of multiple biomarkers. With the aim of reasonable applying these markers to the early detection, accurate diagnosis, and scientific management of kidney disease, thereby mitigating the threat posed by kidney disease to human health.

## 1 Background

**Figure 1:**
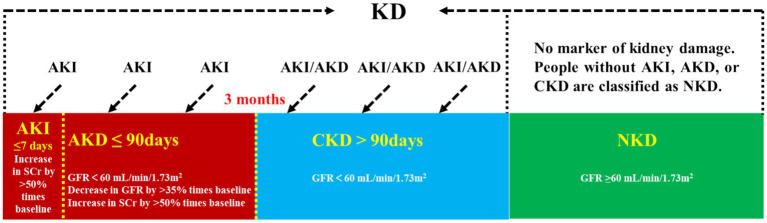
Kidney disease classification. KD, kidney disease; AKI, acute kidney injury; AKD, acute kidney disease; CKD, chronic kidney disease; NKD, no known kidney disease; SCr, serum creatinine; GFR, glomerular filtration rate.

Kidney disease is classified as chronic kidney disease (CKD) or acute kidney disease (AKD) depending on their duration (Figure 1). Acute kidney injury (AKI) is defined as a greater than 50% increase in serum creatinine (sCr) levels within 7 days or a rise to 0.3 mg/dL within 2 days. It is a prevalent condition that affects 10–20% of all hospitalized inpatients and is linked to high rates of morbidity, mortality, and medical expenses (1). In AKI survivors, CKD, heart disease, and other associated comorbidities are more likely to develop. Clinical practice guidelines for AKI (KDIGO) were based on changes in sCr and urine volume. sCr is easily affected by diet, muscle content and other factors; urine volume is also easily affected by urinary tract obstruction, volume status, and other factors. Due to limited sensitivity and specificity, risk stratification systems and models for AKI are rarely clinically validated and accepted (2, 3). And it cannot suggest the etiology of AKI (4). Therefore, the need for early, sensitive, stable, and reliable AKI indicators is still urgent. AKD refers to the period of acute or subacute injury and reduced kidney function that persists for 7 to 90 days following AKI. This interval is a significant phase after the occurrence of AKI, and the management during this time is crucial to the outcome of AKI (2, 5).

CKD denotes a condition where the renal structure or function is abnormal for a period exceeding 3 months. It, characterized by its high prevalence, high disability rate, high medical expenditure, and low awareness, has become a significant global health threat. Global burden of disease studies indicated that in 2017, the prevalence of CKD worldwide was 9.1%, affecting approximately 700 million patients, with one-third of them residing in China and India (6). In the 2023 Sixth National Chronic Disease Prevalence Survey in China, the current proportion of CKD was found to be 8.2%, with a corresponding awareness rate of just 10% (7). Based on the glomerular filtration rate (GFR), kidney disease can be classified into five stages, with stages 3–5 being designated as CKD. During these stages, renal function progressively deteriorates. If left untreated, the disease can advance to stage 5, which is known as end-stage renal disease (ESRD). ESRD, if not addressed promptly, can lead to a myriad of complications and poses a significant risk to life.

In this paper, we reviewed the methods of kidney disease detection and the characteristics of different biomarkers in the diagnosis and progression of kidney disease. Concurrently, we explored the value of combining multiple markers in kidney disease. Thus, our goal is to increase people’s knowledge of kidney disease markers and to choose more sensible and sensitive detection techniques and markers when assessing kidney function.

## 2 The methods of kidney disease detection

There are many diagnostic methods for kidney disease, including renal biopsy and various clinical indicators. Among them, the clinical indicators include urinary formed components, urinary biochemical indicators (such as Cr and urea nitrogen), and urine specific protein markers. The comparison of each index detection method is shown in Table 1.

**Table 1:** Comparison of diagnostic methods for kidney disease.

Methods	Advantages	Disadvantages
Renal biopsy (121, 122)	Is the kidney disease diagnosis gold standard, can diagnose the kidney disease and the type.	It is an invasive test that is not easily accepted by patients; bleeding, nephrectomy, pain, nephrectomy and other complications may occur. Contraindications: bleeding risk, hypertension, small hyperechoic kidneys, anatomical kidney problems, hydronephrosis, solitary kidney, infection and altered mental status.
Urine sediment components (123, 124)	Simple, advanced, and invaluable urine analysis; it serves as an essential tool for assessing patients with AKI and for identifying proteinuria, hematuria, and leukuria in urinalysis.	It is unable to more fully capture the features of the situations. Low accuracy. It is not sensitive to some diseases, such as interstitial nephritis disease, proliferative lupus glomerulonephritis, and acute tubular injury necrosis.
Urinary biochemical (125, 126)	Fast and efficient; wide application; include dipstick urinalysis and dry chemistry urine analyzer.	Semi-quantitative, low sensitivity, high false negative and false positive rates; prone to interference from many exogenous substances, medications, etc.; test results are related to the experience of the operator.
Urine specific protein analysis (22, 39)	It is simple and easy to use, has a broad range of applications, is highly sensitive, and can identify different proteins to pinpoint the location of kidney damage.	The cost is rather high, and more research is still needed to determine the clinical importance of several markers.

## 3 Biomarkers of kidney disease

**Figure 2:**
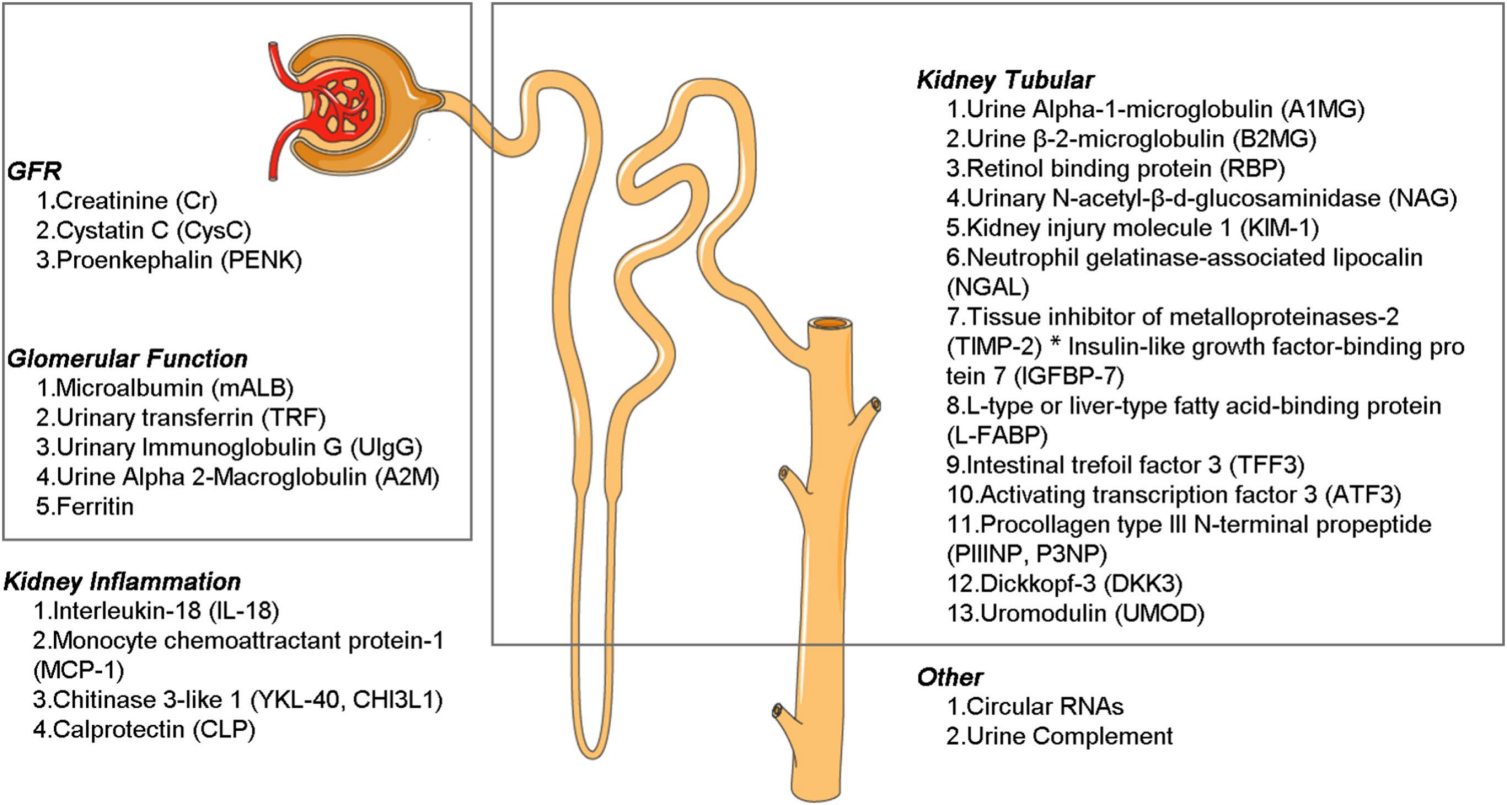
Different types of kidney disease biomarkers.

The diverse biomarkers have different functions, and by the character and concentration of these substances, they enable precise diagnosis of the location and severity of kidney injury. This information is crucial for guiding treatment decisions, evaluating the prognosis of diagnostic and therapeutic interventions, and conducting comprehensive kidney assessments as part of routine health screenings. Broadly speaking, these markers can be categorized based on their anatomic localization and pathophysiological relevance into three main groups: (a) biomarkers that reflect glomerular and tubular (proximal tubule, medullary loop, and distal tubule) function and injury; (b) inflammatory markers; and (c) markers associated with repair and fibrosis (Figure 2).

### 3.1 Glomerular filtration rate

Creatinine (Cr) is the terminal catabolite of creatine and phosphocreatine, and its generation is therefore mainly dependent on muscle mass. Most Cr is eliminated by glomerular filtration; thus, it can be employed to assess kidney function as an endogenous biochemical marker. The international guideline-recommended screening marker for CKD, eGFR, can be calculated for disease staging and classification by measuring sCr and urine Cr. However, filtration indicators like Cr have inadequate sensitivity and specificity for diagnosing kidney disease; it is also raised when there is no kidney damage, but it can also stay the same if the patient has good underlying renal function and the damage is not severe (8–10). Cr is usually detected in urine biochemical analysis together with urea nitrogen and uric acid (Table 2). And it is often used in conjunction with other markers; the diagnostic detection rate of linked disorders is improved (11, 12).

**Table 2:** Main biomarkers of urinary biochemical.

Markers	Molecular weight	Diagnosis of the location	Clinical significance	Related diseases
Urea nitrogen (BUN) (127)	~24KDa	Glomerulus, glomerular filtration, kidney injury	It is one of the markers of glomerular filtration function. The rate of acute kidney damage detection is increased when urinary NAG is detected in combination.	Kidney disease, sepsis
Uric acid (UA) (128, 129)	~168 g/mol	Renal tubules, glomerular filtration, kidney injury	It is one of the markers for detecting kidney injury.	Diabetic kidney disease, CKD, Gout, arthritis

Cystatin C (CysC) is a 13-kDa cysteine proteinase inhibitor protein, which is produced by all nucleated cells. It is freely filtered by the kidney with near-complete reabsorption and catabolism in the proximal tubule and no significant urinary excretion. So urinary CysC can indicate tubular damage. It is most often used as an indicator to assess glomerular filtration function, like Cr. Unlike Cr, serum CysC levels are not influenced by muscle mass, gender, age, body size and composition, or nutritional status. CysC level increases in early diabetes and pre-diabetic diabetic nephropathy (DN). Apart from other biomarkers, CysC is also an independent association with cardiovascular disease, ESRD, and all-cause mortality (13). The usage of CysC is limited by higher detection costs than Cr and standardization problems. Despite the release of an internationally certified Cyc C reference material (ERM-DA471/IFCC) by the International Federation of Clinical Chemistry (IFCC), significant differences still exist between tests from different manufacturers (14). In recent year manufacturers have markedly improved the accuracy and between-method agreement of CysC measurement procedures, which allows for greater confidence in estimated GFR relying on CysC (15).

Proenkephalin (PENK) is produced by proenkephalin A (the precursor molecule of the enkephalin family). Penk is a potential biomarker to measure glomerular function in critically ill patients since it does not bind to proteins in plasma and is exclusively filtered in the glomerulus. PENK may be a more suitable and accurate marker for determining real GFR since it has a greater correlation with measuring GFR (mGFR) than the Cr (16). According to numerous studies, plasma PENK concentrations can be used to forecast clinical outcomes and future renal deterioration in a variety of patients, including those with burns, sepsis, and acute heart failure (17).

### 3.2 Biomarkers of glomerular function

#### 3.2.1 Microalbumin

mALB is a sensitive biomarker for early renal damage, particularly in diabetic and hypertensive nephropathy, and is widely used for diagnosis and monitoring of glomerular diseases (18). mALB elevation may also result from non-glomerular diseases such as urinary tract infections or heart failure, so it cannot be used alone for glomerular disease diagnosis and should be combined with other biomarkers like sCr and CysC for comprehensive evaluation (19). In the 2024 KDIGO CKD Guideline, urinary mALB-to-Cr ratio (UACR) is used to define and categorize CKD. Although albuminuria has been shown to be a crucial diagnostic and prognostic indicator of diabetic CKD, its daily fluctuations have not been sufficiently taken into account. Multiple urine collections for UACR may improve the capacity to monitor changes over time in clinical but may not be necessary for the diagnosis of albuminuria (20). mALB levels correlate with renal prognosis and cardiovascular disease risk, enabling prediction of progression to ESRD and cardiovascular events in patients with CKD (21).

#### 3.2.2 Urinary transferrin

TRF is a 76.5 kDa glycoprotein that has been widely studied as a potential marker of glomerular injury. The negative charge of urinary TRF is less than that of urinary mALB. When the charge barrier of the glomerulus is damaged in the early stage, and urinary TRF is more likely to leak out than urinary mALB. Therefore, transferrin can more sensitively reflect the damage of the glomerular charge barrier. Urinary TRF had a sensitivity of 75% and a specificity of 100% in predicting early DN (22). Compared to mALB, TRF levels may be a more sensitive indicator for identifying glomerular damage and nephropathy in diabetic patients (23). According to reports, over 30% of type 2 diabetes mellitus patients with normal proteinuria had higher urine TRF levels (24).

#### 3.2.3 Urine immunoglobulin G

Immunoglobulin G (IgG) is a protein of high molecular weight (150 kD), which is excreted in large quantities when the permselectivity of the glomerular capillary wall is more severely disrupted. When the glomerulus is seriously damaged, UIgG is generally discharged in large quantities, forming the so-called non-selective proteinuria in clinic. The increase in UIgG reflects the serious damage to the glomerular basement membrane (25). In the 2020 KDIGO guidelines, UIgG was included in risk stratification for membranous nephropathy (UIgG < 250 mg/d is intermediate risk; UIgG > 250 mg/d is high risk). UIgG will worsen tubule interstitial damage in patients with IgA nephropathy (IgAN) whose global hard rate is higher than 25%. It can also indicate the extent of tubule atrophy and interstitial fibrosis (26).

#### 3.2.4 Urine α2-macroglobulin

A key inhibitor of plasma proteinase, A2M is primarily produced by hepatocytes and mononuclear phagocyte cells. It is essential for controlling tissue repair, signal transduction, protease activity, and nutritional maintenance. A wide range of physiological and pathological processes are influenced by A2M, which is also essential for protecting against infections and external toxins. It also regulates hormones, cytokines, and other bioactive lipid factors (27). Studies revealed elevated levels of urinary A2M in patients with DN, indicating that urinary A2M levels rose with the aggravation of the condition (28). A2M also is a regulator of the fibrogenic process in glomerular mesangial cells. Smad3, NFAT5, and FOXP1 were identified as critical regulators of high glucose-induced A2M promoter activation in glomerular mesangial cells (29).

#### 3.2.5 Ferritin

Ferritin is a soluble protein with a molecular weight of 450 kDa, typically made up of 12 “light” chains (Flc) and 12 “heavy” chains (Fhc) (30). Because the glomerulus typically cannot filter the higher molecular weight of ferritin and because there is no filterable free FLC or FHC in the bloodstream, ferritin in urine comes from the kidney (renal tubular epithelial cells). The average concentration of ferritin in normal human urine was 2.2 ug/L, which is only 3% of the level of serum ferritin (31). Therefore, tubulointerstitial lesions brought on by glomerular damage and other CKD—of which renal tubular iron deposition is one of the reasons—are potential causes of elevated urine ferritin levels (32). Studies have indicated that patients with lupus nephropathy (LN) had a considerable rise in urine ferritin levels, with the degree of the increase being correlated with the severity of the disease. Ferritin and urinary ferritin/Cr ratio (UFCR) are a potential biomarker for LN activity (33, 34).

### 3.3 Biomarkers of kidney tubular

#### 3.3.1 Urine *α*-1-microglobulin, urine *β*-2-microglobulin, and retinol binding protein

α-1-microglobulin (A1MG) is a 27-kDa glycoprotein produced by the liver. A1MG levels are very stable in several pathological conditions: no significant changes were seen in the serum of patients with neoplastic diseases, central nervous system disorders, infections, rheumatoid arthritis and other disorders. Altered total A1MG concentrations in plasma or serum are always related to impaired liver or kidney functions (35). Urine A1MG is more crucially used in clinical applications as a sensitive indicator of tubular damage.

*β*-2-microglobulin (B2MG) is a 11.8-kDa polypeptide, which forms the beta chain of the human leukocyte antigen class I molecule. It is present on the surface of most nucleated cells. Most of B2MG is normally eliminated by the kidney via glomerular filtration and subsequent tubular. Extrarenal breakdown and elimination appear to be negligible (36).

Retinol binding protein (RBP), was identified in immunoelectrophoresis in 1961. Only a small portion is eliminated from the urine after being filtered via the glomeruli, with the majority being reabsorbed by the proximal renal tubular epithelial cells (37).

The tubular dysfunction biomarkers, urine A1MG, urine B2MG and RBP, are low-molecular-weight proteins that can be used to evaluate the tubular functions’ potential for reabsorption. A1MG had higher sensitivity and specificity than B2MG in identifying drug-induced kidney damage (38). RBP was a superior option because β2-MG was unstable in acid urine (39). Studies have shown that RBP could be used as reliable or good predictors of diabetic kidney disease (DKD), and the β2MG displayed a weaker diagnostic ability than RBP (40).

#### 3.3.2 Kidney injury molecule 1

KIM-1 is a type 1 transmembrane protein that has a mucin and immunoglobulin domain. In the proximal tubule of the post-ischemic rat kidney, its expression is noticeably elevated (41). In cases of acute or chronic renal damage, KIM-1 expression increases, particularly in the proximal convoluted tubule’s apical membrane. By identifying phosphatidylserine (PS) on the surface of small extracellular vesicles (sEVs), it can also function as a receptor to enhance sEV absorption and exacerbate tubulointerstitial inflammation (TII); this is correlated with the severity of acute and chronic kidney injury (42, 43). There is no Kim-1 in the urine when the kidneys are in health (44). Therefore, KIM-1 is an ideal potential marker for renal injury detection, particularly for proximal tubular (PT) injury early assessment. It is yet unknown how KIM-1 works, but it may possibly be a potential biomarker for neutrophil antibody-associated vasculitis with glomerulonephritis AKI and tubulointerstitial injury (45).

#### 3.3.3 N-acetyl-*β*-d-glucosaminidase

NAG is an enzyme found in the lysosomes of proximal renal tubular cells. Its molecular weight is large enough to prevent it from passing through the usual glomerular basement membrane. It has been shown that an elevated excretion of NAG is more specific for renal tubular disease and indicates active tubular injury (46). There is a report showing that burn patients show persistent NAG elevation during aggressive treatment, indicating that burn patients still have persistent tubular injury after transient AKI (47). The urine NAG/Cr biomarker level has a significant difference between healthy participants and people with type 2 diabetes mellitus (T2DM). It should be measured for the people with T2DM of the first-time diagnosis (48).

#### 3.3.4 Neutrophil gelatinase-associated lipocalin

The lipocalin-2 superfamily includes the tiny protein known as neutrophil gelatinase-associated lipocalin (NGAL). In the kidneys and other human tissues, it is typically expressed at relatively low levels (49). When renal tubular epithelial cells are damaged, it is extensively expressed. As a result, it can be easily found in adult blood and urine shortly after acute renal injury. It has also been demonstrated that urinary NGAL can predict the degree of acute kidney damage following heart surgery as well as the long-term renal outcomes following therapy in an intensive care unit (50). For the diagnosis of AKI, a number of blood and urine NGAL cut-offs have been suggested; however, these cut-offs have not been standardized (51). Dialysis and chronic renal disease patients also have higher levels of NGAL.

#### 3.3.5 Tissue inhibitor of metalloprotease-2 and insulin-like growth factor binding protein 7

TIMP-2 and IGFBP7 are involved in G1 cell cycle arrest and are released in the urine during tubular epithelial cell stress (52). In renal biopsies from nephropathy patients, their expression was significantly elevated, particularly in the renal tubular region: TIMP-2 was mainly detected in the collecting duct, whereas IGFBP7 was mainly detected in the proximal and distal tubules (53). Urine [TIMP-2]*[IGFBP7] is a biomarker for AKI risk assessment and may augment AKI staging for patients with functional criteria for AKI (54). Although it is best utilized in conjunction with other indicators or clinical risk factors, it can also be used to predict a bad prognosis in AKI. It can also be used to forecast a poor outcome in AKI, although preferably in conjunction with other clinical risk factors or markers (55).

#### 3.3.6 Liver-type fatty acid-binding protein

FABPs are a class of low-molecular-weight cytosolic proteins that have between 14,000 and 15,000 homologies. Numerous tissues, such as the intestinal mucosa, the liver, the heart, adipose tissue, the kidney, muscle, and other tissues, contain it. In mammals, at least nine FABPs have been identified. Liver-FABP, or L-FABP, was initially discovered in the liver and is mostly expressed in the kidney’s proximal tubular epithelial cells (56). Urine L-FABP is a potential biomarker for tracking the decline of renal function and early kidney disease identification. Research has indicated that in patients with renal impairment, changes in urine L-FABP levels occur before changes in urinary albumin (57). The urine of patients with AKI, particularly acute tubular necrosis, has higher amounts of L-FABP (58). Kamijo et al. observed in their study of type 2 diabetes patients that increased urine L-FABP is a risk factor for the advancement of DKD and that these levels properly reflect the stage of DKD (59).

#### 3.3.7 Trefoil factor 3

TFF3 is a member of the human trefoil factor family. Under healthy conditions, it’s extensive production in renal tubules. TFF3 may be an important molecule for future studies of kidney disease. Serum and urine TFF3 levels in CKD patients are typically higher than in healthy controls, and they rise significantly as the disease progresses (60). According to a study of kidney transplant recipients, TFF3 levels in their serum and urine increased the day following the surgery and then declined 6 months and a year later. TFF3 has great potential for renal transplant patient monitoring (61).

#### 3.3.8 Activating transcription factor 3

Exosomes are the nano-vesicles released by most of the cells that contain a variety of proteins. and it is an important source of biomarkers. In urine exosomes, ATF3 is one of the key indicators (62). ATF3 plays a crucial role in how cells react to stress in their internal and external environments. Additionally, it plays a major role in physiological and pathological processes such as wound healing, tumor development, cell adhesion, and homeostasis maintenance (63). ATF3 was found earlier than sCr in the urine of patients with AKI, but not in normal persons, sepsis patients without AKI, or patients with CKD. Urinary ATF3 may therefore be a new biomarker for identifying renal tubular cell injury in early AKI and AKI in sepsis (62, 64).

#### 3.3.9 Procollagen type III N-terminal propeptide

Collagen type I and III are the two major proteins involved in extracellular matrix (ECM) remodelling, which leads to the accumulation of fibrotic tissue in several organs, such as the heart or the kidneys. Procollagen type I carboxy-terminal propeptide (PICP) and PIIINP are peptidases that activate two types of collagen, which can indicate the rate of collagen production and be associated with fibrosis in the heart, liver, muscle, renal tubulointerstitium, and others. They can be used to gauge the severity of a number of illnesses and track the progression of fibrosis and tissue healing (65). Research shows PICP is an independent predictor of mortality in ESRD patients who are not yet on dialysis. P3NP levels in the urine may be used as a non-invasive early marker of renal fibrosis, and the ratio of P3NP to Cr can also show how renal fibrosis is progressing in recipients of renal transplants (66, 67).

#### 3.3.10 Dickkopf-related protein 3

DKK-3 is a secreted glycoprotein that is synthesized by stressed tubular epithelia. And *in vitro*, it is highly expressed in mesenchymal cells and mesenchymal progenitor cells (68). DKK3 is secreted in the renal tubular epithelium under stress and leads to tubulointerstitial fibrosis via the Wnt signaling pathway (69, 70). DKK3 was elevated in patients with reduced eGFR (71). In children with CKD, urinary DKK3 can also indicate a short-term risk of renal decline. By identifying individuals who benefit from pharmacological renal protection, such as intense hypertension therapy, it may also allow for customized drug therapy (72). As a consequence, it is considered to be a valuable biomarker for assessing the different causes of renal failure.

#### 3.3.11 Uromodulin

UMOD, or Tamm-Horsfall protein, is the most prevalent protein in healthy people’s urine and is expressed by renal tubular epithelial cells localized in the thick ascending limb of the loop of Henle. The plasma and 24 h urine UMOD are direct markers of Henle’s loop integrity (73). UMOD may indicate the distal renal tubules’ capacity for protein production. Urinary UMOD has been found to be inversely correlated with the progression of CKD, the risk of cardiovascular disease-related death, and the incidence of AKI following heart surgery. These findings may be helpful in determining the risk of death for patients with diabetes and CKD, particularly when it comes to the risk of cardiovascular disease-related death (74). Since UMOD cannot cross through the placental barrier, it can be used to identify AKI in neonates and is also regarded as a biomarker of fetal renal tubular development (75). Recent studies have demonstrated its role as a marker of tubulointerstitial fibrosis, urine UMOD is independently associated with tubulointerstitial fibrosis in both human kidney biopsies and a mouse model of fibrosis (76).

### 3.4 Biomarkers of kidney inflammation

#### 3.4.1 Interleukin-18

A crucial proinflammatory cytokine secreted by macrophages, IL-18 contributes to inflammation and the advancement of renal disease. Renal injury and fibrosis may be lessened by the loss or inhibition of IL-18, which offers a potential therapeutic target for the treatment of renal diseases. CKD is related to the activation of inflammasomes, the production of IL-18, and the stimulation of downstream pro-inflammatory signaling pathways (77, 78). Elevated urine IL-18 levels within 24 h of intensive care unit admission have been independently associated with poor clinical outcomes at 28 days and can offer valuable information for prognostic assessment. They also have some usefulness in predicting the development of AKI (79). In patients who have received a kidney transplant, IL-18 is also a reliable and early indicator of the requirement for dialysis and the recovery of graft function after 3 months (80).

#### 3.4.2 Monocyte chemoattractant protein-1

MCP-1 belongs to the family of chemokines. It plays a crucial part in the pathophysiology of inflammatory nephropathy and promotes monocyte release from the bone marrow, creates chemokine gradients, and guides monocyte migration to inflammatory areas (81). Urine MCP-1 is produced by the kidney rather than as a result of serum MCP-1 filtration (lack of correlation between urinary and serum MCP-1 levels) (82). Urine MCP-1 is a more useful indicator of lupus nephritis activity than blood (83).

#### 3.4.3 Chitinase-3-like protein 1 or YKL-40

YKL-40 is a glycoprotein. There are several cells that can express it besides monocytes, such as neutrophils, fibroblasts, chondrocytes, vascular smooth muscle, endothelial cells, hepatic stellate cells, colon, duct, and airway epithelial cells, and macrophages (84). Similar to MCP-1, urine YKL-40 has been demonstrated to be a marker of inflammation and structural kidney damage. One of the main causes of AKI and CKD development is persistent renal inflammation, and elevated levels of this inflammation have been linked to worsened renal function in hospitalized patients (85). Higher urine levels of YKL-40 and KIM-1 have been associated with an increased risk of all-cause death in people with CKD and diabetes (74). The report is suggests that urine YKL-40, as opposed to urine or plasma NGAL, be employed as a precise and trustworthy biomarker to identify patients at risk of AKI after transplantation (86).

#### 3.4.4 Calprotectin

CLP is a calcium and zinc-binding protein with a relative molecular mass of 36 KD, which is a trimer composed of two heterologous calcium-binding proteins (S100A8 and S100A9). It belongs to the S100 protein family and is produced by neutrophils and monocytes during inflammation (87). Urinary concentration of CLP is substantially increased in intrinsic AKI. And CLP is more effective than NGAL and KIM-1 at differentiating between prerenal and intrinsic AKI, and it would avoid needless biopsies in cases of prerenal disease (88, 89). In CKD patients, CLP is involved in vascular calcification, and CLP in blood can also be used as a biomarker to predict the occurrence of cardiovascular disease. Targeting calprotectin significantly improves vascular calcification in patients with CKD (90). As an acute-phase protein, circulating CLP is significantly correlated with a higher risk of new-onset CKD in the general population (91).

### 3.5 Other

#### 3.5.1 Circular RNA

CircRNA is a unique kind of long non-coding RNA that is distinguished by its circular, covalently closed structure, which resists exonucleases from breaking it down. It is very stable and exhibits tissue and cell type selectivity in its expression pattern. CircRNA is widely distributed in bodily fluids (such as blood, urine, and saliva) and exosomes, which are released by the majority of cell types. As a result, it can be applied as a novel liquid biopsy marker for numerous illnesses (92). Different forms of kidney disease also showed distinct patterns of circRNA expression. The expression of circ-0114427 increased in a mouse model of cisplatin-induced AKI and in cultured renal tubular cells exposed to cisplatin; circactr2 was upregulated in human proximal tubule epithelial cells (HK-2) exposed to high hyperglycemia. Human proximal tubular epithelial cells (HK-2) exposed to elevated glucose showed an upregulation of circACTR2. In both cultivated renal tubular cells exposed to cisplatin and a murine model of AKI caused by cisplatin, circ-0114427 expression was increased (93). CircRNA study in kidney disease is still in early stages, and further research is required to fully understand its potential as a biomarker and molecular therapeutic target.

#### 3.5.2 Complement

The complement system is a part of innate immunity, which is crucial for immunological modulation, pathogen infection protection, and internal environment stability. According to numerous studies, activation or dysregulation of complement has been implicated in the pathophysiology of an increasing number of kidney disease. In 2024, KDIGO (Kidney Disease: Improving Global Outcomes) published the first expert consensus on the role of the complement system in kidney disease (94). Many complement inhibitors that target complement are currently being developed for utilization in treating kidney disease (Table 3).

**Table 3:** Biomarkers of kidney disease and their clinical significance.

Type	Biomarker	Molecular weight	Advantages	Disadvantages
GFR	Creatinine (Cr) (113)	~113 g/mol	It is an endogenous marker and utilized to calculate the GFR. Low cost and widely used worldwide. Assays are well standardized. Non-GFR determinants are well identified.	Influenced by muscle mass, protein intake, and tubular secretion.
GFR	Cystatin C (CysC) (13, 130–132)	~13KDa	It is an endogenous marker and utilized to calculate the GFR. Not affected by race. Provides more information than Cr	More expensive assays. Less standardized assays. Non-GFR determinants are more diverse and some may not yet be fully identified.
GFR	Proenkephalin (PENK) (16, 17, 133)	~33KDa	PENK outperformed sCR in the kidney function assessment and sCR trajectory over time.	There are some conditions that showed an influence on PENK level besides the kidney function, such as traumatic brain injuries, and ischemic or hemorrhagic strokes. Need to further confirm the scientific evidence.
Glomerular Function	Microalbumin (mALB) (11)	~67Kda	Urinary mALB-to-Cr ratio (UACR) is used to define and categorize CKD.	mALB elevation may also result from non-glomerular diseases such as urinary tract infections or heart failure.
Glomerular Function	Urinary transferrin (TRF) (22, 23)	~76KDa	It can reflect the damage of glomerular charge barrier and is more sensitive than ACR.	The majority of TRF research was cross-sectional; therefore, more prospective studies with a larger sample size are required.
Glomerular Function	Urinary Immunoglobulin G (UIgG) (134–136)	~160KDa	UIgG has a higher sensitivity than that of mALB in reflecting changes in renal hemodynamics and inflammation.	IgG is reabsorbed in the tubules. Local production of IgG (e.g., prostate; seminal vesicles) and low-grade urinary tract infections can be additional causes of errors.
Glomerular Function	Urine Alpha 2-Macroglobulin (A2M) (137)	~770KDa	It occurs in the urine when the glomerulus is severely damaged.	In diabetic glomeruli elevation of A2M transcript levels, demonstrating increased local production.
Glomerular function	Ferritin (30, 33, 138)	~450KDa	Urinary ferritin might be a biomarker to detect kidney damage caused by iron toxicity.	Currently, there is limited research and the clinical significance is unclear.
Kidney tubular	Urine Alpha-1-microglobulin (A1MG) (25, 35, 139)	~33KDa	It is one of the markers to evaluate the function of renal proximal tubular. More stable in acidic urine. The tubular reabsorption of A1MG is earlier than that of B2MG, especially in the early detection of DN.	Serum A1MG may be decreased in patients with cirrhosis or hepatitis, and interferes with the diagnosis of renal injury.
Kidney tubular	Urine β-2-microglobulin (B2MG) (36, 140)	~12KDa	It is one of the markers to evaluate the function of renal proximal tubular. Its ratio to Cr can accurately reflect the concentration and dilution function of the kidney and glomerular filtration function.	Instability in urine. Non-renal diseases (e.g., systemic lupus erythematosus, multiple myeloma disease) or lymphocyte diseases can also lead to elevated B2MG.
Kidney tubular	Retinol binding protein (RBP) (37, 39, 141)	~21KDa	URBP play an important role in early prediction of renal impairment. Additionally, it is a marker for predicting renal interstitial fibrosis.	It is synthesized by the liver, and liver diseases (such as hepatitis and cirrhosis) may cause a decrease in serum that interferes with the diagnosis of kidney injury.
Kidney tubular	Urinary N-acetyl-β-d-glucosaminidase (NAG) (46, 142, 143)	~24KDa	It is a marker of acute renal tubular injury. The rate of acute kidney damage detection is increased when BUN is detected in combination.	Some systemic diseases (such as diabetes, hypertension) may indirectly lead to NAG elevation through metabolic abnormalities.
Kidney tubular	Kidney injury molecule 1(KIM-1) (41, 144–146)	~90KDa	It is a potential marker of AKI. RBP excretion was not affected by posture, circadian rhythm, or short-term diet.	Some inflammatory diseases, such as sepsis, may indirectly lead to KIM-1 elevation and interference should be excluded. Further validation of standardized testing methods is needed.
Kidney tubular	Neutrophil gelatinase – associated lipocalin (NGAL) (147–149)	~25KDa	It is a potential marker of AKI. The diagnostic accuracy of NGAL for AKI risk was excellent.	Increased with urinary tract infection. And current assays cannot distinguish NGAL monomers from homodimers.
Kidney tubular	Tissue inhibitor of metalloproteinases-2 (TIMP-2) and Insulin-like growth factor-binding protein 7 (IGFBP-7) (52, 150–152)	~21KDa and ~33KDa	It is a potential marker of AKI. [TIMP-2]•[IGFBP7] testing is particularly useful within the first 72 h of ICU admission.	Some decisions lack scientific evidence. Less consensus was present on when to test.
Kidney tubular	L-type or liver-type fatty acid-binding protein (L-FABP) (153–155)	~14KDa	It is a potential marker of AKI.	Liver production increased in polycystic kidney disease an sepsis.
Kidney tubular	Intestinal trefoil factor 3 (TFF3) (60, 61, 156)	~6.7KDa	It is a potential marker of AKI. It can be used to evaluate the renal development of preterm neonates.	TFF3 as a prognostic biomarker of AKI and CKD needs to fully consider the possible effects of ethnicity, age, sex and the type of kidney disease.
Kidney tubular	Activating transcription factor 3(ATF3) (63, 64)	~22KDa	It is a potential marker of AKI.	Lack of studies with large sample sizes.
Kidney tubular	Procollagen type III N-terminal propeptide (PIIINP, P3NP) (157–160)	~42KDa	It is a biomarker of the early stages of tubulointerstitial fibrosis.	Urine PIIINP was not associated with CVD events and death in kidney transplant recipients.
Kidney tubular	Dickkopf-3(DKK3) (68, 161–163)	~38KDa	It is a potential marker of AKI. uDKK3 emerged as a predictor of interstitial fibrosis	Elevated urine DKK3 was not confirmed, which is associated with a higher risk of mortality.
Kidney tubular	Uromodulin (UMOD) (75, 164, 165)	~85KDa	It is a biomarker for tubular mass and function. It is a predictor of interstitial fibrosis.	Urine UMOD levels measured are affected by centrifugation, vortexing, storage conditions, and polymerization of urine UMOD, thereby affecting quantification.
Kidney inflammation	Interleukin-18 (IL-18) (77, 79, 166)	~18KDa	It is a potential marker of AKI.	Increased with inflammation of many tissues.
Kidney inflammation	Monocyte chemoattractant protein-1 (MCP-1) (81, 85)	5-20KDa	It seems to be a key player in the pathogenesis of inflammatory kidney disease.	MCP-1 levels may be influenced by other inflammatory factors and may vary in AKI and CKD.
Kidney inflammation	Chitinase 3-like 1 (YKL-40, CHI3L1) (85, 86, 167)	~40KDa	It is associated with fibroblast accumulation and interstitial renal fibrosis.	Its pathophysiological mechanism is still unknown in AKI, and there are still few validated cutoffs.
Kidney inflammation	Calprotectin (CLP) (91, 147, 168)	~36KDa	It is a promising early marker for evaluating AKI. Related to vascular calcification in patients with CKD.	It has limited value in predicting the differentiation of inflammatory and non-inflammatory CKD, CKD prognosis, and the risk of new CKD.
Other	Circular RNAs (92, 169)	NA	It is involved in the pathogenesis and progression of many kidney disease and is a new therapeutic target and biomarker of nephropathy.	Need more prospective trials to ascertain their relationship to important clinical outcomes.
Other	Urine Complement (94, 170)	25 ~ 390KDa	It is involved in the pathogenesis and progression of many kidney disease and plays an important role in monitoring the disease. It provides a therapeutic target for new drug development.	Need more research to validate existing and future diagnostic and prognostic tools.

## 4 Novel biomarkers

Increasing studies have shown that omics (including genomics, transcriptomics, proteomics, metabolomics, epigenomics, and more) was used for the identification of various biomarkers in renal diseases.

### 4.1 Genomics and metagenomes

According to the study by Nadia Ayasreh et al., MUC1, UMOD, HNF1B, REN and SEC61A1 have been found to be the pathogenic genes of autosomal dominant tubulointerstitial kidney disease, suggesting that these genes play an important role in the maintenance of renal function (95). The number of genome-wide association studies (GWAS) of kidney function has increased, from single-ethnic to cross-ethnic studies, from tens of thousands to millions of people, and the number of loci found has also increased. In a 2019 study, researchers identified 308 loci with 760,000 participants for the discovery group that explained 7.1% of the eGFR variation, validation with another group of 280,000 participants and the addition of the two groups resulted in the identification of 264 loci (96).

The human body is known to have a diverse and abundant collection of bacteria known as the microbiota (97). Furthermore, a number of diseases, including obesity, type 2 diabetes, hepatic steatosis, intestinal bowel disorders, and many cancers, are now thought to be associated with the gut microbiota (98). Mei Z et al. conducted a comprehensive study of the T2DM microbiome, analyzing 8,117 shotgun metagenomes. And they found that dysbiosis in 19 phylogenetically diverse species was associated with T2DM (false discovery rate < 0.10), for example, enriched *Clostridium bolteae* and depleted *Butyrivibrio crossotus*. These microorganism changes were potentially underlying T2DM pathogenesis (99). Linh HT et al. have identified an increase in *Klebsiella oxytoca* genes in the blood of DKD patients. And its copies correlated with higher sCr and BUN levels together with lower eGFR in DKD patients (100). Through microbial metabolites, microbes can communicate with their hosts. The latest publication showed that gut microbiota (like *Lactobacillus* species) could affect membranous nephropathy (MN) through tryptophan-produced indole derivatives that engage host receptors (101). A study by Zeng Y et al. found that both DKD patients and mice had significantly lower levels of indole-3-propionic acid (IPA), a metabolite generated from tryptophan, which was negatively correlated with impaired renal function. Supplementing with IPA improved albuminuria and strengthened the glomerular filtration barrier (102).

### 4.2 Proteomics and metabolomics

Proteomics uses mass spectrometry and bioinformatics analysis techniques to enable a comprehensive analysis of the proteins in a sample. Using untargeted proteomic profiling of circulating proteins, Md Dom ZI et al. identified three elevated protective proteins-fibroblast growth factor 20, angiopoietin-1, and tumor necrosis factor ligand superfamily member 12-that were associated with protection against progressive renal decline and progression to ESRD (103). Another study by Niewczas MA et al., screened 194 circulating inflammatory proteins for KRIS (kidney risk inflammatory signature), which consists of 17 proteins enriched in tumor necrosis factor-receptor superfamily members, a marker of inflammatory processes involved in the development of ESRD in diabetes (104). Catanese L et al. analyzed the proteomics data of urine samples and identified a peptidomic classifier for renal fibrosis containing 29 peptide fragments corresponding to 13 different proteins. And urinary proteomics analysis can serve as a non-invasive tool to evaluate the degree of renal fibrosis, which allows repeated measurements in contrast to kidney biopsy (105). These proteins may serve as biomarkers to stratify diabetic individuals according to risk of progression to ESRD and might also be investigated as points to new therapeutic targets of DN.

Metabolomics is an analytical study technique that shows the byproducts of biological processes in living things using high-throughput analysis. These metabolites consist of a variety of compounds, including sugars, lipids, and amino acids (106). Metabolomics has considered an efficient tool in the search for biomarkers that are critical for precision health approaches and improved diagnostics. A study by Wang et al. demonstrates that CKD is associated with phosphatidylcholine (PC) metabolism disorders by conducting ultra-high-performance liquid chromatography, and the administration of *Rheum officinale* can improve impaired kidney function and aberrant PC metabolism in CKD rats (107).

### 4.3 Integrating multi-omics

The integration of multi-omics data, including the genome, transcriptome, and metabolome, will play a key role in biomarker identification, uncovering intracellular molecular networks, and complex disease mechanisms. Wang et al.’s integrated metabolite–protein network delivers a thorough view of the “disease interactome,” offering mechanistic insights for improved clinical management (108). Si S et al.’s integrated analysis of plasma proteome and transcriptome data identifies 32 potential therapeutic targets for CKD, kidney function, and specific CKD clinical types. These proteins included 12 proteins with prior Mendelian randomization support and 20 novel causal proteins that have not been previously reported (109).

## 5 Biomarker combination

In most cases, detecting biomarker combinations is more efficient than using just one sign. As a result, a reasonable combination of several biomarkers can more properly estimate the degree of kidney health, injury location, and the type of disease (Table 4) (110–112).

**Table 4:** Summary of biomarker combination.

Study	Biomarker combination	Related type of kidney disease	Description
Alexandra K Lee et al. (171)	NGAL, IL-18, YKL-40, KIM-1, MCP-1, A1MG, B2MG, UMOD, iPTH and iFGF-23	CKD	By combining kidney tubule health biomarkers using an unsupervised approach, this study identified multiple factors that defined unique aspects of kidney tubule health.
Buddhi N T W Fernando et al. (172)	A1MG, KIM-1, and RBP	CKD	A 3-marker signature panel comprising A1M, KIM1, and RBP4 was identified to represent the best minimum marker combination for differentiating all CKD categories, including CKDu, from healthy controls with an overall sensitivity of ≥0.867 and specificity ≥0.765.
Claudio Bazzi et al. (173)	Urinary/serum IgG and Cr	IgA nephropathy	In treated patients, progression prediction was increased by FEIgG/SG and sCr in combination (0 versus 89%, increased to 0 versus 100%, FEIgG: fractional excretions of IgG).
Claudio Bazzi et al. (174)	Urinary IgG and A2M	Idiopathic focal segmental glomerulosclerosis (FSGS)	Low and high risk groups of FEIgG and A2M/C in combination had very high predictive value of sustained remission and ESRD in response to therapy. ESRD prediction increased to 0% versus 89% (*p* < 0.0001) and prediction of remission increased to 83% versus 11% (*p* = 0.008) in patients with both FE IgG and α2m/C below or above their respective cutoffs
Yukun Zhou et al. (37)	eGFR, A1MG and URBP	DKD	Drew ROC based on eGFR, A1MG and URBP to evaluate the diagnostic effectiveness of the model. The diagnostic model achieved a high accuracy with an AUC of 0.987, which indicates great performance.
Deyuan Zhang et al. (175)	Urinary TRF, IgG, NGAL, and TNF-α	DN	Study calculated the area under the receiver operating characteristic curves (area under the curve) and found that urinary IgG (0.894), NGAL (0.875), TRF (0.861), TNF-α (0.763), and the combination of urinary TRF + IgG + TNF-α + NGAL (0.922) showed good diagnostic value for early-stage DN.
Kinga Musiał et al. (176)	KIM-1, IL-18, and NGAL	Kidney Injury	The RF Classifier achieved the AUROC of 0.8333, accuracy of 80.00%, positive predictive value of 0.8667, and sensitivity of 0.8000. The contributions of KIM-1, IL-18, and NGAL to the prediction in this model were comparable (33.73, 32.77, and 33.5%, respectively).
Ying Zhang et al. (177)	Cys C,β2-MG, urinary NGAL, and A1MG	AKI	Values for NGAL gave the best diagnostic performance, with an AUC of 0.865. The sensitivity and specificity of combined diagnosis of AKI in asphyxiated neonates with the above 4 indicators were 0.974 and 0.506, respectively.
Jianchao Ma et al. (178)	Serum Cr, sCys C and uNAG	AKI	The first clinical model included the APACHE II score, sCr, and vasopressor used at ICU admission. This model could predict AKI with reasonable certainty (AUC-ROC = 0.784). Adding sCysC and uNAG to this model improved the AUC to 0.831.
Lei Lei et al. (179)	KIM-1, NGAL and Cys C	AKI	Urinary KIM-1, urinary NGAL, serum Cys C, and the combined detection factor serving as a screening index for AKI secondary to decompensated cirrhosis had sensitivities of 63.4, 68.3, 80.2 and 89.1%, respectively and specificities of 81.6, 72.1, 74.9, and 95.8%, respectively.
Daisuke Katagiri et al. (180)	L-FABP and NAG	AKI	Of 77 patients, 28 patients (36.4%) developed AKI after surgery. Area under the curve of ROC [AUC-ROC] for L-FABP at 4 h 0.72 and NAG 0.75. Combined these 2 biomarkers can detect AKI with higher accuracy than either biomarker measurement alone (AUC-ROC 0.81).
Tao Sun et al. (181)	A1MG, L-FABP and IGFBP7	AKI	The area under the curve (AUC) of the U-AKIpredTM (combined with A1MG, L-FABP and IGFBP7) as a predictor of AKI within 12 h, was 0.802 (95% CI: 0.771–0.833, *p* < 0.001) in the training set and 0.844 (95% CI: 0.792–0.896, *p* < 0.001) in validation cohort.
Casey M Rebholz et al. (140)	eGFRcr, eGFRcys (Cr or Cys C-based eGFR), and 1/B2MG	ESRD	Using the average of declines in the three markers, >30% decline conferred higher ESRD risk than that for eGFRcr alone (HR, 31.97 [95% CI, 19.40–52.70; *p* = 0.03] vs. eGFRcr).

Cr is the most often used marker in combination with others. Two crucial endogenous biomarkers for eGFR are Cr and CysC. The accuracy of eGFR is also increased by combining the two markers, particularly in cases where Cr and CysC are inconsistent (113). Urinary Cr can also be used to calculate the ratio with other markers, “correct” the quantitative results of other markers (such as mALB, NGAL, NAG, etc.), and reduce the impact of variables like urine concentration on the results (33, 46, 114, 115). The most important is the albumin-to-Cr ratio (ACR), a quick, easy, and precise indicator of the body’s albumin excretion. Its main purpose is to categorize albuminuria in cases of CKD. The cause and mechanism of AKI can also be determined by combining biomarkers of renal injury (e.g., uNGAL) with markers of renal filtration function (e.g., SCr, CysC) (116). For instance, NGAL levels may be influenced by underlying infections however if levels of NGAL were elevated along with elevations of KIM-1 and LFABP the diagnostic likelihood of AKI would be enhanced (117).

In addition to normal marker association studies, Frank Bienaim et al. use LASSO logistic regression analysis to examine 30 urine biomarkers. They find multiple possible biomarker combinations that indicate the rapid advancement of CKD (118).

Table 4 displays the evolution of a few kidney disease biomarker combinations.

## 6 Discussion and future directions

A prevalent issue all around the world is kidney disease. Therefore, there are many markers for the diagnosis of kidney disease. Under specific conditions, these markers have a strong relationship with the diagnosis, management, and prognosis of kidney disease (such as AKI, CKD, lupus nephritis, nephrotoxicity, renal transplantation, DN, hypertensive nephropathy, etc.). The results of them may differ due to variations in the environment, interfering factors, and detecting methods. Many new biomarkers have been discovered and validated, but not all of them are specific for a specific kidney disease. At the same time, the specificity of these biomarkers may vary depending on the clinical situation of the patient. A broad understanding of the unique characteristics of biomarkers, as well as their sensitivity and specificity in different types of kidney disease, is needed. Finally, there is a lack of uniform testing standards and cutoff values for these markers, although some have been approved by the FDA (e.g., Kim-1, TIMP-2*Igfbp-7) (119). Therefore, the introduction of these biomarkers into medical practice remains challenging. On the other hand, the combination of biomarkers often leads to better detection performance, so exploring the combination of more markers is also an important research direction to obtain more accurate diagnostic results (like Frank Bienaim et al.).

The field of genomics has advanced quickly since 1986, propelling the growth of fields such as transcriptomics, proteomics, metabolomics, epigenomics, and more. Using omics technology to analyze the body fluids of patients with kidney disease, followed by machine learning tools and pathway analysis, differs from traditional research methods in that it allows us to rapidly screen a large number of disease-related candidate markers and potential therapeutic targets (120). However, the application of omics in kidney disease faces two significant challenges: (a) Due to the limited detection technology, few omics-screened markers can be comprehensively and in-depth verified, so they are still difficult to apply to clinical practice; (b) The transformation of biomarkers from basic research to clinical application is a complex process. It requires technological breakthroughs and large-scale population studies; (c) In the application of clinical practice, it is necessary to strengthen the education and training of clinicians, and develop user-friendly tools, so that clinicians can interpret the results easily and conveniently.

With the increase of detection methods, markers and OMICS technology to generate more and more large data sets, artificial intelligence (AI) has become an important tool to analyze these complex data. The application of AI in kidney diseases is gradually increasing and shows great potential. AI technologies, especially machine learning and deep learning, can be used to analyze complex clinical data, including biochemical indicators, imaging data, and electronic medical records to help clinicians better diagnose kidney diseases. AI technology can also use patients’ genetic information, biomarkers and drug response data to customize treatment plans and achieve precision diagnosis and treatment. However, the application and promotion of AI face many problems such as data privacy and security issues, transparency and interpretability of algorithms, complex regulatory environment, standardization and quality control. Therefore, the economic factors and practical difficulties involved in integrating these technologies into existing health care systems may be greater than we think.

However, it will be essential to clinical diagnosis and treatment, including focused therapy and exact diagnosis, to improve patient diagnosis, treatment, and prognosis as technology develops and research becomes more comprehensive.

## 7 Conclusion

Kidney function can be examined using a variety of methods and markers, each with pros and cons of its own. Numerous putative biomarkers for renal health have demonstrated promise in enhancing the clinical management of kidney disease and are implicated in the pathophysiological process of kidney injury. It has been demonstrated that these biomarkers can identify and detect kidney injury at an early stage, as well as forecast the course, severity, and long-term risk of death associated with the disease. With the study of the combination of markers and the discovery of new markers, the detection of kidney disease will be more rapid and accurate.
